# Validation of the Arabic version of the binge eating scale and correlates of binge eating disorder among a sample of the Lebanese population

**DOI:** 10.1186/s40337-019-0270-2

**Published:** 2019-12-01

**Authors:** Rouba Karen Zeidan, Chadia Haddad, Rabih Hallit, Marwan Akel, Karl Honein, Maria Akiki, Nelly Kheir, Souheil Hallit, Sahar Obeid

**Affiliations:** 1 Faculty of Public Health, Lebanese University, Fanar, Lebanon; 2 INSPECT-LB: Institut National de Santé Publique, Epidemiologie Clinique et Toxicologie, Beirut, Lebanon; 3 CERIPH: Center for Research in Public Health, Pharmacoepidemiology Surveillance Unit, Faculty of Public Health, Lebanese University, Fanar, Lebanon; 4 Psychiatric Hospital of the Cross, P.O. Box 60096, Jall-Eddib, Lebanon; 5 Univ. Limoges, UMR 1094, Neuroépidémiologie Tropicale, Institut d’Epidémiologie et de Neurologie Tropicale, GEIST, 87000 Limoges, France; 6 Faculty of Medicine and Medical Sciences, Holy Spirit University of Kaslik (USEK), Jounieh, Lebanon; 7 School of Pharmacy, Lebanese International University, Beirut, Lebanon; 8 Faculty of Pedagogy, Holy Family University, Batroun, 5534 Lebanon; 9 Faculty of Arts and Sciences, Holy Spirit University (USEK), Jounieh, Lebanon

**Keywords:** Binge eating disorder, Psychometrics properties, Depression, Body dissatisfaction, Emotions, Anxiety

## Abstract

**Objectives:**

To test the psychometric properties of the Arabic version of the Binge Eating Scale (BES), a self-questionnaire assessing binge eating, in a sample of the Lebanese population. The secondary objective was to evaluate factors associated with binge eating.

**Methods:**

This cross-sectional study, conducted between January and May 2018, enrolled 811 adult participants from all districts of Lebanon. The BES was administered to study its psychometric properties. The sample was divided into two separate samples (405 for sample 1 and 406 for sample 2). An exploratory factor analysis was executed on Sample 1, followed by a confirmatory factor analysis on Sample 2 using the structure obtained in Sample 1. Three hierarchical stepwise linear regressions were conducted to assess factors associated with binge eating.

**Results:**

The factor analysis suggested a two-factor structure for the BES explaining a total of 41.4% of the variance. All items could be extracted from the list. The internal consistency of the measurement was adequate (Cronbach’s alpha = 0.86). The confirmatory factor analysis revealed an adequate fit to the model with satisfactory Maximum Likelihood Chi-Square/Degrees of Freedom (χ^2^/df), Steiger-Lind RMSEA, Joreskog GFI, and AGFI. Higher BMI, depression, anxiety, emotional eating, greater body dissatisfaction, and more pressure from media to lose weight were associated with higher binge eating. Higher expressive suppression facet score was associated with lower binge eating.

**Conclusion:**

The Arabic version of BES could be a useful tool for screening and assessing the binge eating behaviors in clinical practice and research. Also, being dissatisfied with one’s body size, having a history of sexual abuse, family history of binge eating, increased depressive/anxiety symptoms, and lower self-esteem seem to be associated with higher BES scores.

## Plain English summary

The Binge Eating Scale in its Arabic version is a suitable instrument to screen for binge eating among the Lebanese population. Some factors (body dissatisfaction, having a history of sexual abuse, a family history of binge eating, greater depressive/anxiety symptoms, and lower self-esteem) seem to be associated with higher binge eating.

## Introduction

Binge eating disorder (BED) is an eating disorder (ED) characterized by recurrent episodes of binge eating (BE) without subsequent compensatory behaviors, such as self-induced vomiting or over-exercising [1]. Diagnosis of BED according to the Diagnostic and Statistical Manual of Mental Disorders, Fifth Edition (DSM-5), is based on the occurrence of at least one BE episode per week for three consecutive months; these episodes are characterized by the consumption of larger amounts of food in a short period when compared to the typical amounts for most people under similar circumstances, accompanied by a sense of loss of control over eating and a marked distress during these episodes [2]. A large study, pooling results from different community surveys done across 14 countries (high, upper-middle, and lower-middle income countries), revealed an average lifetime prevalence estimate of 1.9% [0.2–4.7%] for BED, making it the most common ED [3, 4]. Previous reviews showed that ED most frequently afflict young Western females within high-income and industrialized Western Europe and North America, but do also occur in diverse countries and cultures worldwide [5, 6]. While there is a stabilization - even a lowering -of the incidence rates of eating disorders in Caucasian North American and Northern European groups, increased rates appear in Arab and Asian countries [7]. In fact, as Lebanon and other countries in the Middle Eastern and North African region continue to develop and undergo epidemiologic transition and cultural change, the burden of ED in these countries is rising [8, 9].

### Binge eating correlates

The disorder was found to be more common in the population suffering from obesity [3], and it has been associated with several psychiatric (anxiety, depression, obsessive–compulsive disorder) and physical comorbidities (diabetes, hypertension) [3, 6, 10]. Previous findings showed that adverse childhood experiences (i.e. sexual/physical abuse, other parental problems), parental depression, negative self-evaluation, vulnerability to obesity, and repeated exposure to negative comments about shape, weight, or eating were associated with BED. These findings support the hypothesis that risk factors associated with BED are similar to those with psychiatric disorders and obesity [11]. Furthermore, researchers have found BED to share characteristics with substance use and addictive disorders [12, 13]. In addition to other similarities, BED and substance use can both be induced by stress and major life events, childhood trauma, neglect, and abuse [14–16].

### Arabic countries and Lebanon

BED research remains limited in the Arab-speaking countries, despite overweight and obesity being important public health issues. Older studies done in Egypt [17, 18] first showed evidence for BE, without reporting any prevalence rates. Other findings revealed that 82% of females and 76% of males (mean age = 20 years) had experienced at least one BE episode [19]. In Jordan, results showed that 16.9% of females (mean age = 13) reported BE and 1.8% were considered as suffering from BED [20]. The prevalence of BE in Saudi Arabia females was 68.8% [21], whereas it ranged between 24 to 36% in the United Arabic Emirates [22].

In Lebanon, previous research revealed that 21.2% of one-university students were vulnerable to developing an ED, whereas 11.4% had already been diagnosed with an ED. The same paper showed that anxiety was the most important associated factor with ED, followed by stress, body image and depression [23]. Another Lebanese study published in 2017, done on patients suffering from ED and comparing BED to anorexia nervosa and bulimia nervosa, concluded that outpatients were mostly single, female, young adults coming from middle and high socio-economic backgrounds, suffering from severe ED symptoms and depression [24].

### The binge eating scale (BES)

Due to the high prevalence of BE, its comorbidities and outcomes, there was a need to establish instruments for its measurement. The BES, developed by Gormally et al. [25], is an interesting tool both in terms of evaluation and monitoring of these patients, because it can be used for the purposes of screening, evaluation of severity or monitoring of the disorder. The BES has been found to demonstrate a very good internal consistency (between 0.85 and 0.90) and a good construct validity [25, 26].

The scale has previously been validated in many populations, including but not limited to, French [27], Italian [28], Spanish [29], Persian [30] and Malay [31]. The BES was found to have good validity and internal consistency in the original normative samples, consisting of overweight women seeking behavioral treatment for obesity [25]. A study done by Imperatori et al. that evaluated the dimensionality and psychometric properties of the Italian version of the BES had found that the scale had good internal consistency (α = 0.89), with a moderate mean inter-item correlation [28]. A study done among a sample of 1008 women from the general population confirmed that the BES presents a one-dimensional factorial structure, with very good construct reliability and convergent validity, and with a very good retest reliability [32]. In the French version, one factor was obtained that explained 61% of the total variance [27]. Timmerman had also found a good test-retest reliability r = 0.87 for the total BES score. However, its factor structure is still controversial. Although Gormally et al. originally proposed a two-dimensional structure, dividing the items into cognitive and behavioral BE, results on its dimensionality are contradictory. In a Mexican [33] and a Malay study [31], results showed a two-factor structure through exploratory factor analyses; they also found the unidimensional structure to be relevant as well. A study done on Portuguese women found the BES to have a good fit for a one-factor structure using a confirmatory factor analysis [32]. This was also supported by a French study done in non-clinical and clinical samples using an exploratory factor analysis [27]. A recent Spanish study targeting a sample of Spanish college students reported that a one-factor model fit the data best [34].

To our knowledge, no publication has reported the validation and the psychometric properties of the BES in the Arabic-speaking population. In addition, there is a need for more evidence about the factor structure of the scale in non-clinical samples, because most research has tested the validity of BES in specific populations (sufferers of overweight/obesity and bariatric surgery patients). Also, no study has been previously done on the Lebanese general population in order to study BED and its correlates when compared to healthy individuals. Hence, the aim of this work was to test the psychometric properties of an Arabic version of the BES, by establishing the factorial structure, its internal consistency and its construct validity in a sample of the Lebanese population. Another objective was to study BED’s associated sociodemographic and clinical factors.

## Methods

### Participants

This study was cross-sectional, conducted between January and May 2018. Out of 1000 distributed questionnaires, 811 (81.1%) were completed and collected. Enrollment of participants was done using a proportionate random sample from all Lebanese Mohafazat (Beirut, Mount Lebanon, North, South and Bekaa). Each Mohafaza is divided into Caza (stratum). In each Caza, two villages were randomly selected from the list of villages provided by the Central Agency of Statistics in Lebanon. Participants were randomly selected from each village. In each selected village, the questionnaire was distributed randomly to the households, based on random sampling technique to select the included house. Houses were assigned identification numbers and randomized according to an online software, Research Randomizer (www.randomizer.org). All members in the household, if eligible, were invited to participate in the study; those who accepted our invitation were asked to fill out the questionnaire. The same methodology was used in previous papers [35–38].

Prior to participation, individual subjects were briefed on the study objectives and methodology, and were assured of the anonymity of their participation. Individuals agreeing to participate in the study were then asked to read through and sign off on a written, informed consent form. Individual participants had the right to accept or refuse participation in the study with no financial compensation provided in exchange for individual participation. All participants above 18 years of age were eligible to participate. Excluded participants were those suffering from a clinical mental impairment affecting cognition and their ability to comprehend participation in the study and/or their ability to answer the questionnaire. The sample was divided into two separate samples for the validation of the BES (Study 1: *n* = 406 for the exploratory analysis; Study 2: *n* = 405 for the confirmatory analysis; therefore, 406/16 = 25 participants per item were included in study 1 and 405/16 = 25 per item in study 2 as well). However, the whole sample (*n* = 811) was used to study the factors correlated with BE.

The mean age of the participants in the first sample was 27.96 ± 11.83 years, with 67.6% females. The majority (73.3%) had a university level of education, single (66.1%), with a low monthly income (76.4%). Almost all participants drank caffeine (90.5%), 31.7% were smokers and 3.1% were drank alcohol. The majority practice physical activities (63.6%). More than half of the participants had normal weight (52.4%), 27.2% were overweight and only 11.1% suffered from obesity, with a mean BMI of 24.10 ± 4.98 Kg/m^2^. The majority of the participants (82.8%) had no binge eating, 15.9% had moderate BE and 1.3% had severe BE.

In sample 2, the mean age of the participants was 27.19 years, 65.4% were females, 67.8% were single, and 79.2% had low monthly income. The majority had a university degree (73.1%) and 61.3% practice physical activities. More than half of the participants had normal weight (53.8%), 26.5% had overweight and only 12.4% suffered from obesity, with a mean BMI of 24.60 ± 5.55. No significant difference was found between the two samples (*p* > 0.05) (Table 1).

**Table 1:** Characteristics of the study sample

	Total	Sample 1	Sample 2	p- value
	Frequency (%)	Frequency (%)	Frequency (%)	
Gender				
Male	270 (33.5%)	131 (3.4%)	139 (34.6%)	0.518
Female	536 (66.5%)	273 (67.6%)	263 (65.4%)	
Marital status				
Single	533 (67.0%)	263 (66.1%)	270 (67.8%)	0.395
Married	230 (28.9%)	120 (30.2%)	110 (27.6%)	
Widowed	11 (1.4%)	7 (1.8%)	4 (1.0%)	
Divorced	22 (2.8%)	8 (2.0%)	14 (3.5%)	
Education level				
Primary	24 (3.1%)	11 (2.8%)	13 (3.3%)	0.760
Complementary	61 (7.8%)	34 (8.7%)	27 (6.9%)	
Secondary	125 (15.9%)	60 (15.3%)	65 (16.6%)	
University	574 (73.2%)	288 (73.3%)	286 (73.1%)	
Monthly income				
No income	340 (45.1%)	168 (44.4%)	172 (45.7%)	0.140
< 1000 USD	247 (32.8%)	121 (32.0%)	126 (33.5%)	
1000–2000 USD	117 (15.5%)	56 (14.8%)	61 (16.2%)	
> 2000 USD	50 (6.6%)	33 (8.37%)	17 (4.5%)	
Smoking				
Yes	246 (30.8%)	127 (31.7%)	119 (29.8%)	0.572
No	554 (69.2%)	274 (68.3%)	280 (70.2%)	
Alcohol				
Yes	32 (4.2%)	12 (3.1%)	20 (5.4%)	0.124
No	724 (95.8%)	372 (96.9%)	352 (94.6%)	
Caffeinated beverages				
Yes	721 (90.0%)	362 (90.5%)	359 (89.5%)	0.646
No	80 (10.0%)	38 (9.5%)	42 (10.5%)	
Practicing sport activities				
Yes	490 (62.4%)	248 (63.6%)	242 (61.3%)	0.501
No	295 (37.6%)	142 (36.4%)	153 (38.7%)	
BMI categories				
Underweight	65 (8.3%)	36 (9.3%)	29 (7.3%)	0.733
Normal	417 (53.1%)	204 (52.4%)	213 (53.8%)	
Overweight	211 (26.9%)	106 (27.2%)	105 (26.5%)	
Obese	92 (11.7%)	43 (11.1%)	49 (12.4%)	
	Mean ± SD	Mean ± SD	Mean ± SD	
Age (in years)	27.59 ± 11.76	27.96 ± 11.83	27.19 ± 11.68	0.354
BMI (Kg/m^2^)	24.35 ± 5.28	24.10 ± 4.98	24.60 ± 5.55	0.182

### Minimal sample size calculation

Comrey and Lee suggested that a minimum of 10 observations per item is necessary in order to avoid computational difficulties [39]. Since the BES questionnaire contains 16 questions, a minimal sample of 160 patients was needed to conduct an exploratory factor analysis.

### Procedure

Data collection was performed through personal interviews with participants by trained clinical psychologists, independent of this study, who clinically evaluated the level of psychiatric illness to exclude those who have psychiatric problems.

### Materials

The questionnaire used during the interview was in Arabic, the native language of Lebanon. The first part assessed the sociodemographic details and other characteristics of the participants (i.e. age, gender, monthly income, education level, details about alcohol and caffeine intake, drug addiction, smoking status, and the physical activity). The monthly income was divided into four levels: no income, low income < 1000 USD; intermediate income 1000–2000 USD; and high income > 2000 USD. Afterward, the monthly income was categorized in three groups: low (no income with income < 1000 USD), intermediate (1000–2000 USD) and high (> 2000 USD) income, since the majority of the participants were university students and they have no real income. The heights and weights of participants were measured to calculate the Body Mass Index (BMI) (kg/m^2^). The BMI was classified into four categories: underweight (< 18.5 kg/m^2^), normal (18.5.24.9 kg/m^2^), overweight (25.0.29.9 kg/m^2^), and obese (30.0 kg/m^2^) [40]. The Total Physical Activity Index was calculated by multiplying the intensity, duration, and frequency of daily activity, with higher scores indicating better physical activity.

The second part of the questionnaire consisted of behavioral practices of certain eating habits among participants. Questions about eating disorders were identified from previous articles [41–43]. Questions included: “Do you take your weight daily?”, “Do you follow a diet to lose weight?”, “Do you exercise to lose weight?”, “Do you take diet pills to lose weight?”, “Do you take laxatives or vomit to lose weight?”, “Do you starve yourself to lose weight?”

### Binge eating scale (BES)

The BES was originally developed to identify participants with binge eating within people suffering from obesity [25]. It does not specify a time frame and presents a series of differently weighted statements for each item, from which respondents select the statement that best describes their attitudes and behaviors. This yields a continuous measure of BE pathology of 0–46. Scores of ≥27 have conventionally served as a cutoff value for identifying the presence of severe BE and 17 as a cutoff value for mild or no BE [44]. The BES has good test-retest reliability (r = 0 .87, *p* < 0 .001). The Arabic version can be found at the end of this manuscript (Additional file 1: Appendix 1).

### Body dissatisfaction subscale of the eating disorder inventory-second version (EDI-2)

In the present study, the body dissatisfaction score was measured from the eating disorder inventory (EDI-2) subscale since the body dissatisfaction could cause restraint eating which in turn causes susceptibility to loss of control over eating. The scale assesses the levels of dissatisfaction with the overall body shape and specific body parts. The body dissatisfaction subscale consists of nine items, measured in 4-point Likert scales, ranging from 0 (sometimes, rarely, never) to 3 (always). Five questions were reversed while doing the score calculation. The total score was calculated by summing the nine items. The total score ranged from 0 to 27. Higher scores are indicative of greater body dissatisfaction [45]. In this study, the Cronbach’s alpha was 0.779.

### Self-esteem scale

The Rosenberg Self-Esteem10-item scale is used to assess beliefs and attitudes regarding general self-worth by using 4-point scales ranging from 1 (strongly disagree) to 4 (strongly agree). Five questions (3, 5, 8, 9, and 10) were reversed while doing the score calculation. Higher scores indicated a more negative self-esteem [41]. In this study, the Cronbach’s alpha was 0.759.

### Perceived stress scale (PSS)

The questions in the PSS ask about feelings and thoughts during the last month [42]. The PSS is a ten-item scale with answers ranging from never (0) to almost always (4). Items 4, 5, 7, and 8 are reversed items. The total score is calculated by summing the 10 items with higher scores indicating more perceived stress [42]. In this study, the Cronbach’s alpha was 0.709.

### HAM-A scale

The Hamilton Anxiety Rating Scale (HAM-A), recently validated in Lebanon [43], consists of 14 symptom-defined elements, and targets both psychological and somatic symptoms. Each item is scored on a basic numeric scoring of 0 (not present) to 4 (severe). Higher scores indicating higher anxiety. In this study, the Cronbach alpha’s was 0.912. The Arabic version can be found at the end of this manuscript (Additional file 1: Appendix 2).

### HAM-D scale

The Hamilton Scale for Depression (HAM-D), validated in Lebanon [46], is a multiple item questionnaire used to provide an indication of the severity of depression and is widely used in research. The 17-item version was used in this study, with each item rated on either a 3- or 5-point scale and summed to obtain the total score. Higher scores indicated higher depression. In this study, the Cronbach’s alpha was 0.879. The Arabic version can be found at the end of this manuscript (Additional file 1: Appendix 3).

### Emotion regulation questionnaire (ERQ)

The ERQ is used to measure respondents’ tendency to regulate their emotions in two ways: (1) Cognitive Reappraisal and (2) Expressive Suppression. The cognitive reappraisal facet is a way of managing and controlling attention and cognitively changing the meaning of emotionally stimulating stimuli. It is considered a healthy emotion regulation strategy [47]. The expressive suppression involves inhibition of emotionally expressive behavior, thereby changing the emotional impact of a situation [48]. It is considered a less healthy emotion regulation strategy [48]. A 10-item scale ranging from 1 (strongly disagree) to 7 (strongly agree). Items 1, 3, 5, 7, 8, 10 make up the Cognitive Reappraisal facet and items 2, 4, 6, 9 make up the Expressive Suppression facet. Each facet’s scoring is kept separate. The higher the scores, the greater the use of the emotion regulation strategy [49]. In this study, the Cronbach’s alpha values for the Cognitive Reappraisal facet and for the Expressive Suppression facet were 0.764 and 0.658 respectively.

### Emotional eating scale (EES)

The EES scale is composed of twenty-five items, with three derived subscales: anger, anxiety and depression. Participants rate the extent to which certain feelings lead to the urge to eat, using a five-point Likert scale ranging from 0 (no desire to eat) to 4 (an overwhelming urge to eat). Higher scores indicate a reliance on using food to help manage emotions [50]. The original EES scale had a good test-retest reliability (r = 0.79, *p* < 0.001) and an acceptable internal consistency Cronbach alpha = 0.81 [51]. In this study, the Cronbach’s alpha was 0.957.

### State adult attachment measure (SAAM)

The SAAM measures 3 different aspects of adult attachment: security, anxiety, and avoidance. A secure attachment style is characterized by the confidence in the emotional balance in times of distress and need [52]. The attachment anxiety is characterized by a perceived failure to handle threats, which intensifies need for interpersonal closeness, love, and support [53]. The attachment avoidance is characterized by discomfort with interpersonal intimacy, hesitancy to trust others, and preventing the emotions evoked by rejection by others [54]. It consists of 21 Likert style questions ranging from 1 (strongly disagree) and 7 (strongly agree). Higher scores indicate high features of attachment [55]. In this study, the Cronbach’s alpha was 0.827.

### Dutch restrained eating scale

The Dutch Restrained Eating Scale, recently validated in Lebanon [35], is a ten-item scale that assesses the frequency of dieting behaviors by using a five-point Likert scale, ranging from 1 (never) to 5 (always). The score for this scale was obtained by dividing the total items score by the total number of items. A higher score would indicate a higher degree of restrained eating. In this study, the Cronbach’s alpha was 0.928. The Arabic version can be found at the end of this manuscript (Additional file 1: Appendix 4).

### Translation procedure

The forward and backward translation method was conducted in all the scales except for the HAMD, HAMA and Dutch restrained eating scales (the Arabic versions of these scales can be found in Additional file 1: Appendices 2, 3 and 4). The forward translation was done by a single bilingual translator, a health professional familiar with the terminology of the scales, whose native language is Arabic and is fluent in English. An expert committee formed by healthcare professionals and a language professional verified the Arabic translated version. A backward translation was then performed by a native English speaker translator, fluent in Arabic, and unfamiliar with the concepts of the scales. All translators were informed of the purpose of the study prior to translation. The back-translated English questionnaire was subsequently compared to the original English one, by the expert committee, aiming to discern discrepancies and to solve any inconsistencies between the two versions. Revisions of problematic questions were communicated with the translators involved for version updating. The process of forward-back translation was repeated until all ambiguities disappeared.

### Statistical analysis

The SPSS software version 23 was used to conduct data analysis. Descriptive analyses were done using counts and percentages for categorical variables, mean, standard deviation for continuous measures. As for the reduction of data, an exploratory factor analysis was first executed to detect groups of factors that are reported together and are associated with BE in the sample. After confirming sample adequacy with the Kaiser–Meyer–Olkin (KMO) index and Bartlett’s Chi-square test of sphericity, factors (groups of items) were extracted using a principal component analysis method and a promax rotation since factors were correlated. We retained factors with an Eigenvalue higher than one; we confirmed their adequacy with a Scree plot, taking into account interpretability of the results. Items with factor loading > 0.4 were considered as loading on a factor. We also checked the reliability using Cronbach’s alpha values for different factors and the total scale.

Second, a confirmatory factor analysis was carried out in Sample 2 using the maximum likelihood method for discrepancy function to assess the structure of the instrument. We also reported several goodness-of-fit indicators: the Relative Chi-square (χ^2^/df), the Root Mean Square Error of Approximation (RMSEA), the Goodness of Fit Index (GFI) and the Adjusted Goodness of Fit Index (AGFI). The value of χ^2^ divided by the degrees of freedom (χ^2^/df) has a low sensitivity to sample size and may be used as an index of goodness of fit (cut-off values:< 2–5). The RMSEA tests the fit of the model to the covariance matrix. As a guideline, values of< 0.05 indicate a close fit and values below 0.11 an acceptable fit. The GFI and AGFI are Chi-square-based calculations independent of degrees of freedom. The recommended thresholds for acceptable values are ≥0.90 [56].

The Student T-test was used to compare continuous variables in two groups. Pearson correlation was used for linear correlation between continuous variables. For categorical variables, the Chi-square and Fisher exact tests were used. The ANOVA F tests were used when comparison involved three or more groups. A post-hoc analysis, Bonferroni test was used to compare the mean difference between groups. Three hierarchical stepwise linear regressions were conducted, taking the BES as the dependent variable. All variables that showed a *p* < 0.1 in the bivariate analysis were considered as important variables to be entered in the model in order to eliminate potentially confounding factors as much as possible. These three models were built by adding variables to the previous model at each step in order to determine that the newly added variables would improve the proportion of explained variance of the dependent variable by the model (improve in adjusted R^2^). In the first model the sociodemographic were considered as predictor factors, in the second model the eating behaviors were added, and in the third models the emotion scales were added. The stepwise method was used to simultaneously remove variables that were weakly correlated to the dependent variable. Thus, the final variables kept in the model better explain the distribution. A *P*-value of less than 0.05 was considered significant.

## Results

### Study 1

#### Exploratory factor analysis

None of the BES items were removed. All items could be extracted from the list since no items over-correlated to each other (r > 0.9), had a low loading on factors (< 0.3) or because of a low communality (< 0.3). The factor analysis for the BES was run over the first sample (Total *n* = 406). The BES items converged over a solution of two factors that had an Eigenvalue over 1, explaining a total of 41.40% of the variance. A Kaiser-Meyer-Olkin measure of sampling adequacy of 0.925 was found, with a significant Bartlett’s test of sphericity (*p* < 0.001). According to the promax rotated matrix, the first factor accounted for 34.59% of the variance and the second factor accounted for 6.81%. Table 2 displays the items and factor loadings for the factors. The first factor, which seems to index behavioral manifestations of BED, had strong loadings on 12 items. The second factor, which seemed to index feelings/cognitions, had high loadings on the remaining four items. Moreover, a Cronbach’s alpha of 0.862 was found for the full scale, 0.826 for Factor 1 and 0.682 for Factor 2.

**Table 2:** Promax rotated matrix of the Arabic version of the Binge Eating Scale

BES Items	Factor loadings	
	Factor 1	Factor 2
BES 4	0.750	
BES 15	0.677	
BES 10	0.619	
BES 9	0.588	
BES 13	0.586	
BES 5	0.581	
BES 11	0.553	
BES 7	0.552	
BES 16	0.544	
BES 2	0.516	
BES 8	0.508	
BES 3	0.433	
BES 1		0.877
BES 6		0.775
BES 14		0.664
BES 12		0.475
Cronbach alpha	0.826	0.682
Percentage of variance explained	34.59%	6.81%

Cronbach alpha of the whole scale = 0.862

Factor 1: Behavioral manifestations; Factor 2: Feelings/Cognitions

#### Correlation factors

Table 3 displays the correlation factors between each item of the BES and the whole scale. The correlation factors ranged between 0.459 and 0.660 for an individual item. To note that all factors were highly significantly correlated with the whole scale with *p* < 0.001 for all of them.

**Table 3:** Correlation for each item and the total score of the Binge eating scale (BES)

Item	Pearson Correlation coefficient^a^	*p*-value
BES 4	0.620	< 0.001
BES 15	0.579	< 0.001
BES 10	0.660	< 0.001
BES 9	0.559	< 0.001
BES 13	0.570	< 0.001
BES 5	0.526	< 0.001
BES 11	0.648	< 0.001
BES 7	0.616	< 0.001
BES 16	0.575	< 0.001
BES 2	0.584	< 0.001
BES 8	0.548	< 0.001
BES 3	0.602	< 0.001
BES 1	0.562	< 0.001
BES 6	0.583	< 0.001
BES 14	0.629	< 0.001
BES 12	0.584	< 0.001

^a^Corrected item-total correlations were reported

### Study 2

#### Confirmatory factor analysis

A confirmatory factor analysis was run on sample 2 (*n* = 405), using the two-factor structure obtained in Sample 1. The following results were obtained: the Maximum Likelihood Chi-Square = 257 and Degrees of Freedom = 104, which gave a χ^2^/df = 2.4. For non-centrality fit indices, the Steiger-Lind RMSEA was 0.12 [0.104–0.155]. Moreover, the Joreskog GFI equaled 0.799 and AGFI equaled 0.706.

### Bivariate analysis conducted on the whole sample

A significantly higher mean of BES was found in participants with primary education compared to university education (13.04 vs. 8.65, *p* = 0.022). A significantly higher mean of BES was found in patients following a diet (10.75 vs. 8.23, *p* < 0.001), patients who exercised to lose weight (10.26 vs. 8.20, *p* < 0.001), patients who vomited or took laxatives to lose weight (13.37 vs. 8.51, *p* < 0.001), taking diet pills (13.00 vs. 8.57, p < 0.001), starving self to lose weight (12.20 vs. 8.19, p < 0.001), weighing daily (10.75 vs. 8.58, *p* = 0.005) compared to those who don’t follow these eating habits. Also, a significantly higher mean of BES was found in participants that have been insulted (12.40 vs. 8.53, *p* < 0.001), experienced physical abuse (11.26 vs. 8.80, *p* = 0.014) and sexual abuse (15.37 vs. 8.74, *p* = 0.003), felt pressure from media to lose weight (12.80 vs. 8.18, *p* < 0.001), and have a family history of eating disorders (11.86 vs. 8.17, p < 0.001) compared to those who do not agree with these statements.

In addition, more BE was significantly associated with more body dissatisfaction (r = 0.245, p < 0.001), more restrained eating (r = 0.096, *p* = 0.008), higher BMI (r = 0.200, p < 0.001), higher perceived stress (PSC score) (r = 0.194, p < 0.001), higher anxiety (HAMA) (r = 0.344, p < 0.001), higher depression (HAMD) (r = 0.399, p < 0.001), higher state adult attachment scale-avoidance (r = 0.083, *p* = 0.021), and higher emotional eating (EES scale) (r = 0.259, p < 0.001). However, higher emotional regulation cognitive reappraisal facet, expressive suppression facet (r = − 0.113, p = 0.003 and r = − 0.095, *p* = 0.013 respectively), and a more secure adult attachment (r = − 0.173, p < 0.001) were significantly associated with less BE (Table 4). No significant association was found between BES and gender (*p* = 0.758).

**Table 4:** Bivariate analysis of the factors associated with the Binge Eating Scale (BES) score

	BES	*p*-value
	Mean ± SD	
Education level^+^		
Primary	13.04 ± 9.36	0.022
Complementary	8.65 ± 6.86	
Secondary	9.95 ± 8.25	
University	8.65 ± 7.43	
Monthly income		
Low	8.69 ± 7.44	0.459
Intermediate	9.64 ± 8.84	
High	9.26 ± 7.75	
Dieted to lose weight (past 30 days)		
Yes	10.75 ± 7.74	< 0.001
No	8.23 ± 7.47	
Exercised to lose weight (past 30 days)		
Yes	10.26 ± 7.72	< 0.001
No	8.20 ± 7.47	
Vomited or taken laxatives to lose weight (past 30 days)		
Yes	13.37 ± 7.29	< 0.001
No	8.51 ± 7.53	
Taken diet pills to lose weight (past 30 days)		
Yes	13.00 ± 7.69	< 0.001
No	8.57 ± 7.53	
Starving self to lose weight (past 30 days)		
Yes	12.20 ± 8.20	< 0.001
No	8.19 ± 7.30	
Daily weighing		
Yes	10.75 ± 7.92	0.005
No	8.58 ± 7.53	
Receiving comments from the family concerning losing weight		
Yes	10.90 ± 8.03	< 0.001
No	8.12 ± 7.32	
Have you been insulted		
Yes	12.40 ± 8.46	< 0.001
No	8.53 ± 7.34	
Have you been physically abused		
Yes	11.26 ± 8.68	0.014
No	8.80 ± 7.53	
Have you been sexually abused		
Yes	15.37 ± 10.26	0.003
No	8.74 ± 7.44	
Have you been in a bad romantic relationship		
Yes	10.44 ± 8.10	0.001
No	8.38 ± 7.38	
Family history of eating disorders		
Yes	11.86 ± 8.08	< 0.001
No	8.17 ± 7.33	
Pressure from TV, magazine to lose your weight		
Yes	12.80 ± 8.07	< 0.001
No	8.18 ± 7.31	
	Pearson correlation coefficient	*p*-value
Body dissatisfaction score	0.245	< 0.001
Restrained eating scale	0.096	0.008
Body Mass Index	0.200	< 0.001
Perceived stress scale	0.194	< 0.001
Anxiety	0.344	< 0.001
Depression	0.399	< 0.001
ERQ cognitive reappraisal facet	−0.113	0.003
ERQ expressive suppression facet	−0.095	0.013
State Adult attachment scale-security	−0.173	< 0.001
State Adult attachment scale-avoidance	0.083	0.021
EES scale	0.259	< 0.001

*ERQ* Emotional Regulation Questionnaire, *EES* Emotional Eating Scale

^+^One-way analysis of variance (ANOVA): post hoc analysis for education level: primary vs. complementary (13.04 vs. 8.65, *p* = 0.111); primary vs. secondary (13.04 vs. 9.95, *p* = 0.449); primary vs. university (13.04 vs. 8.65, *p* = 0.04); complementary vs. secondary (8.65 vs. 9.95, *p* = 1.000); complementary vs. university (8.65 vs. 8.65, *p* = 1.000); secondary vs. university (9.95 vs. 8.65, *p* = 0.536)

### Multivariable analysis conducted on the whole sample

The results of a first linear regression, considering the BES to be the dependent variable and the sociodemographic as independent variables, showed that a primary level of education compared to illiteracy (Beta = 4.203) was associated with more BE.

A second linear regression, taking the BES as the dependent variable and the opinion about eating habits as independent variables, showed that a higher BMI (Beta = 0.252), starving self to lose weight (Beta = 1.618), pressure from media to lose weight (Beta = 3.036), sexual abuse (Beta = 4.991), receiving comments from the family concerning losing weight (Beta = 1.162) and family history of eating disorders (Beta = 1.582) were associated with more BE.

A third linear regression, taking the BES as dependent variable and the scales and opinion about eating habits as independent variables, showed that higher BMI (Beta = 0.185), higher depression (Beta = 0.298), higher anxiety (Beta = 0.070), higher emotional eating (Beta = 0.072), greater body dissatisfaction (Beta = 0.198) and greater pressure from media to lose weight (Beta = 2.010), were associated with higher BE. Higher expressive suppression facet (Beta = − 0.108) was associated with lower BE (Table 5).

**Table 5:** Multivariable analysis

	Unstandardized Beta	Standardized Beta	*p*-value	Confidence interval	
				Lower Bound	Upper Bound
Model 1: Linear regression taking the Binge Eating Scale (BES) as dependent variable and the sociodemographic characteristics as independent variables.					
Primary vs illiterate* level of education	4.203	0.093	0.009	1.039	7.376
Variables entered: Education level.					
Model 2: Linear regression taking the Binge Eating Scale (BES) as dependent variable and the opinion about eating habits as independent variables.					
Pressure from TV, magazine to lose your weight (yes vs no*)	3.036	0.151	< 0.001	1.522	4.549
Body Mass Index (BMI)	0.252	0.173	< 0.001	0.149	0.356
History of sexual abuse (yes vs no*)	4.991	0.103	0.005	1.505	8.478
Receiving comments from the family concerning losing weight	1.162	0.071	0.061	−0.052	2.376
Starving self to lose weight (past 30 days) (yes vs no*)	1.618	0.083	0.029	0.167	3.069
Family history of eating disorders (yes vs no*)	1.582	0.083	0.030	0.153	3.012
Variables entered = BMI, education level, Exercised to lose weight (past 30 days), Dieted to lose weight (past 30 days), Vomited or taken laxatives to lose weight (past 30 days), Taken diet pills to lose weight (past 30 days), Starving self to lose weight (past 30 days), Daily weighing, Receiving comments from the family concerning losing weight, Have you been insulted, Have you been physically abused, Have you been Sexually abused, Have you been in a bad romantic relationship, family history of eating disorders, Pressure from TV, magazine to lose your weight.					
Model 3: Linear regression taking the Binge Eating Scale (BES) as dependent variable and the eating disorders and emotion scales and opinion about eating habits as independent variables.					
Depression	0.298	0.298	< 0.001	0.216	0.380
Body dissatisfaction	0.198	0.157	< 0.001	0.103	0.292
Emotional eating	0.072	0.192	< 0.001	0.046	0.098
Body Mass Index (BMI)	0.185	0.131	< 0.001	0.082	0.288
Pressure from TV, magazine to lose your weight (yes vs no*)	2.010	0.102	0.005	0.610	3.410
Anxiety	0.070	0.094	0.025	0.009	0.131
Emotional regulation expressive suppression facet	−0.108	−0.072	0.038	−0.210	−0.006
Variables entered in the model: BMI, education level, Exercised to lose weight (past 30 days), Dieted to lose weight (past 30 days), Vomited or taken laxatives to lose weight (past 30 days), Taken diet pills to lose weight (past 30 days), Starving self to lose weight (past 30 days), Daily weighing, Receiving comments from the family concerning losing weight, Have you been insulted, Have you been physically abused, Have you been Sexually abused, Have you been in a bad romantic relationship, family history of eating disorders, Pressure from TV, magazine to lose your weight, Body dissatisfaction score, Restrained eating scale, Perceived stress scale, HAMA, HAMD, ERQ cognitive reappraisal facet, ERQ expressive suppression facet, State Adult attachment scale and EES scale					

*Reference group

## Discussion

Although there are numerous studies on BE behavior worldwide, studies on this behavior in Lebanon remain limited. Therefore, this cross-sectional study aimed to evaluate the psychometric properties of an Arabic version of the BES and to determine the correlates of BED among a sample of the Lebanese population. The results demonstrated that the Arabic version of this tool had a very good internal consistency and a good construct validity. Our results also showed that higher BMI, starving self to lose weight, feeling pressure from media to lose weight, a history of sexual abuse, a family history of eating disorders, higher depression and anxiety scores, higher emotional eating, and greater body dissatisfaction were associated with higher BE scores. A low monthly income compared to no income and a higher expressive suppression facet score were associated with lower BE scores.

### Validation of the BES

The factor structure of the Arabic version revealed two main factors, in line with the Malay study [31]. Also, another study done among a sample of bariatric surgery candidates on the replication and evaluation of BES found that a two-factor model improved significantly the model fit, supporting the presence of a higher-order severity factor accounting for a significant amount of variance [57]. The internal consistency of our results was similar to the original scale [25] and to the other versions (Cronbach alpha ranged from 0.85 [30] to 0.93 [27]).

All BES items positively correlated above 0.5, indicating good predictive value. This was similar to a study done by Grupski et al. that found nearly half of the items correlated positively [58], but conflicted with the study done by Greeno et al., which found very low correlations for most items [44]. The inconsistency in results between the studies might be due to methodological differences and/or sample characteristics. The results obtained in Sample 1 were confirmed later on a second sample, which further solidify the validation of the scale in its Arabic form. The findings of this study demonstrate that the Arabic version of the BES is a valid scale for the assessment of BED the Lebanese general population. This easy-to-administer 16-item scale can be used to provide relevant information about the symptoms and the severity of the BED.

### Binge eating correlates

The results of our study showed that increased depression scores were associated with higher scores on the BES. In fact, comorbid depression has been found in 30–50% of patients suffering from BE [59, 60]. The association between these two disorders has been the subject of many studies and predicts symptoms of BE in white women [61], middle school students [62] and adolescent girls [63]. In a recent meta-analysis, previous authors [55, 64] have shown a reciprocal relationship between depression and BED; in fact depression can be a risk factor for BED, or a result of it. This dual relationship would emphasize the presence of common predisposing factors between the two disorders. Another possible explanation presented in previous studies is that exposure to a food with a pleasant taste is able to cause the activation of brain regions involved in feelings of reward in patients with a high level of negative emotions (such as depression) [65].

Moreover, the multivariable analysis indicates that higher anxiety was associated with more pronounced BE, in line with previous studies [66–69]. The comorbidity rate between BED and anxiety disorders is high, with a lifetime prevalence of 37% among patients suffering from BED [70]. In fact, anxiety disorders are the second most common comorbidity in BED subjects [70]. On the one hand, some studies suggest that anxiety may be secondary to overeating [71]; some genetic data has shown that people with BED have an increased risk of anxiety symptoms, regardless of BMI [72]. On the other hand, retrospective studies have shown that anxiety has been identified as a risk factor for various eating disorders, including BED [73, 74]; more specifically, anxiety disorders generally precede eating disorders, with early onset during childhood [75, 76]. Whether anxiety is a risk factor for BED or a consequence, the literature suggests that BE and anxiety can be linked through common vulnerability factors such as distal stress (for example, during childhood), proximal stress, and childhood stressors (teasing and bullying) [76].

More body dissatisfaction was also associated with greater BE, according to our results. In addition to the risk factors, the triggering and sustaining factors of the BED have been identified and integrated into a recent Tuschen-Caffier and Hilbert model [77]. Based on this model, the authors pointed out that there are different external and internal stressors (relationship conflicts, exposure to food, impulsivity, low self-esteem, tensions) that can trigger BE. In addition, they add that over concentration on weight and shape, as well as body dissatisfaction, are risk factors for the etiology, maintenance and relapse of BED. This model is in line with other studies [78, 79]. Other studies have shown that subjects with BED exhibited negative emotions and had more negative body-related cognitions when examining their own bodies than healthy controls [80, 81]. This disturbed perception of body size and shape may be related to a biased treatment of information, which is now considered an important factor in the development and maintenance of body image disruption in the pathology of the BED [82, 83]. Therefore, this erroneous treatment of information will be an important aspect of psychotherapeutic work in BED subjects [84, 85].

In our study, higher expressive suppression facet was associated with lower BE. Strategies for regulating inappropriate negative emotions (such as disappointment, suffering, loneliness, etc.) have been shown to play a role in the appearance and maintenance of BED [86, 87]. More particularly, people with BED tend to suppress and ruminate on their undesirable emotions, resulting in an increase in psychopathological beliefs and symptoms [88, 89]. Emotional eating was found to be positively related to BE. In fact, overeating just for emotional reasons and cravings may turn quickly into BED [90].

According to our results, a positive and significant correlation was found between a history of sexual abuse and BED, in line with several studies [91, 92]. A correlation was found between traumatic events and eating disorders used as a means of self-management of feelings and experiences related to trauma. Specifically, previous findings [91, 93] have found that 30% of children with an eating disorder have been sexually abused, with BED, in particular, being linked to trauma as a means of self-protection. The cycle of frenzy/overeating behaviors seems to reduce awareness of thoughts and emotions as a way of evading negative emotions that accompany traumatic experiences [94].

As for BMI, our results showed that a higher BMI was associated with higher BED. Previous findings [95] have focused on the significant correlation between obesity and BED and hypothesize that these conditions may potentially contribute to one another and/or exacerbate each other. The co-existence between obesity and BED is of concern due to the medical and psychosocial risks [96].

As for pressure from media, our results indicated a positive correlation between BE and pressure from media to lose weight. Few studies exist specifically on BED and media, but different studies have shown a direct relationship between media exposure, media pressure, eating disorders, body dissatisfaction, and negative affect [97].

Finally, results of our study indicate that a family history of eating disorders was associated with higher BE. BED was found to aggregate strongly within families, which may reflect genetic influences [98, 99]. Also, a population-based twin study found a considerable heritability estimate (41%) for the BED, thus supporting the heritable nature of this syndrome [72]. Although family and twin studies have suggested the role of genetics in BED, gene studies have not confirmed the involvement of a particular gene or genetic pathway. Both genetic and environmental factors are now hypothesized to work together in order to influence the risk for eating disorders. Studies have not yet reached a definitive measure of the extent of relative importance of each component due to the difficulty in statistically distinguishing between the genetic and environmental factors involved in the association [100].

### Limitations

There are many limitations in this study. First, we relied on participants to provide us with information on BE, depression, anxiety, emotion regulation, body dissatisfaction and others using self-report questionnaires. Second, the study has a cross sectional design and causality cannot be proved. Third, although the sample was randomly selected across Lebanese regions, the majority was young, single, with a university degree, which hinders the generalizability of the results. Fourth, test-retest reliability was not assessed. Finally, many scales used were not validated in Lebanon. Nonetheless the authors believe that their findings are noteworthy, since they are consistent with other recent studies.

## Conclusion

The results showed that the Arabic version of the BES could be used as an appropriate measure for assessing BE behaviors in clinical practice and research. The findings suggest that the scale is bi-factorial. The study also found that having higher BES scores is associated with being dissatisfied with one’s body size, having a history of sexual abuse, a family history of BE, increased depressive/anxiety symptoms, and lower self-esteem.

Comprehensive treatments should address the psychological antecedents and consequences of this behavior, as they are critically important to the syndromal nature of BED. It is in this context that the role of mental health professionals is essential in providing interventions for long-term BE episodes and dealing with negative emotions and related psychological factors. Moreover, this study suggests putting into action certain behaviors aimed at preventing individuals from engaging in excessive food consumption: 1) an increase and improvement in family and relational interactions, 2) the promotion of a positive body image, and better self-esteem by professionals and in media 3) prevention of depression and anxiety. These recommendations must be incorporated into intervention programs on eating behavior. Psychologists, counselors, and nutritionists should also work together to improve people’s eating habits, nutritional status, and mental health.

## Supplementary information

**Additional file 1:** **Appendix 1.** The Arabic version of the Binge Eating Scale. **Appendix 2.** The Arabic version of the Hamilton Anxiety Rating Scale (HAM-A). **Appendix 3.** The Arabic version of the Hamilton Scale for Depression (HAM-D). **Appendix 4.** The Arabic version of the Dutch Restrained Eating Scale.

## Data Availability

The authors do not have the right to share any data information as per their institutions policies.

## Abbreviations

AGFI: Adjusted Goodness of Fit Index
ANOVA: Analysis of variance
BE: Binge eating
BED: Binge eating disorder
BES: Binge Eating Scale
BMI: Body Mass Index
DSM-5: Diagnostic and Statistical Manual of Mental Disorders, Fifth Edition
DSM-IV: Fourth Edition of the Diagnostic and Statistical Manual of Mental Disorders
ED: Eating disorder
EDI-2: Body dissatisfaction subscale of the Eating Disorder Inventory-second version
EDNOS: Eating disorder not otherwise specified
EES: Emotional eating scale
ERQ: Emotion Regulation Questionnaire
GFI: Goodness of Fit Index
HAM-A: Hamilton Anxiety Rating Scale
HAM-D: Hamilton Scale for Depression
KMO: Kaiser–Meyer–Olkin
PSS: Perceived Stress Scale
RMSEA: Root Mean Square Error of Approximation
SAAM: State adult attachment measure
