# Supplementary material for: Validation of the Arabic version of the binge eating scale and correlates of binge eating disorder among a sample of the Lebanese population
Source: J Eat Disord. 2019 Dec 1;7:40. doi: 10.1186/s40337-019-0270-2 (PMC6885321; doi:10.1186/s40337-019-0270-2)
Supplement: Supplementary file 1 — Additional file 1: Appendix 1. The Arabic version of the Binge Eating Scale. Appendix 2. The Arabic version of the Hamilton Anxiety Rating Scale (HAM-A). Appendix 3. The Arabic version of the Hamilton Scale for Depression (HAM-D). Appendix 4. The Arabic version of the Dutch Restrained Eating Scale. [file 40337_2019_270_MOESM1_ESM.docx]

**o**

**Appendix 1.**

**The Arabic version of the Binge Eating Scale**

فيما يلي مجموعات من العبارات المرقمة. اقرأ جميع العبارات في كل مجموعة وضع علامة على العبارة الأفضل التي تصف الطريقة التي تشعر بها تجاه المشاكل التي لديك والتي تسيطر على سلوك الأكل الخاص بك.

# 1

ا. لا أشعر بالوعي الذاتي حول وزني أو حجم جسمي عندما أكون مع الآخرين.

ب. أشعر بالقلق حول كيف أتطلع إلى الآخرين، لكنها عادة لا تجعلني أشعر بخيبة أمل مع نفسي.

ج. لدي الوعي الذاتي بالنسبة لمظهري ووزني مما يجعلني أشعر بخيبة أمل مع نفسي.

د. أشعر جدا بالوعي الذاتي حول وزني وكثيراً ما أشعر بالخجل الشديد والاشمئزاز من نفسي. أحاول تجنب الاتصالات الاجتماعية بسبب الوعي الذاتي.

# 2

ا. ليس لدي أية صعوبة في تناول الطعام ببطء بالطريقة الصحيحة.

ب. على الرغم من أنني أبدو وكأني ألتهم الأكل، فإنني في نهاية المطاف لا أشعر بالتُخمة بسبب الأكل أكثر من اللازم.

ج. في بعض الأحيان، أميل إلى تناول الطعام بسرعة وبعد ذلك، أشعر بالشبع بشكل غير مريح.

د. لدي عادة بلع الطعام دون مضغه حقا. عندما يحدث هذا، عادة ما أشعر بأنني محشوّ بشكل غير مريح لأنني أكلت أكثر من اللازم.

# 3

ا. أشعر بالقدرة على التحكم بدوافع الأكل عندما أريد ذلك.

ب. أشعر أنني فشلت في السيطرة على أكلي أكثر من أي شخص عادي.

ج. أشعر بالعجز تماما عندما يتعلق الأمر بالشعور بالتحكم بدوافع الأكل.

د. بسبب شعوري بالعجز عن السيطرة على رغباتي الغذائية، أصبحت يائسا جدا بالنسبة لقدرتي على السيطرة على هذه الرغبات.

# 4

ا. ليس لدي عادة تناول الطعام عندما أشعر بالملل.

ب. أنا أحيانًا أتناول الطعام عندما أشعر بالملل، ولكن في كثير من الأحيان أستطيع إلهاء نفسي وعدم التفكير بالطعام.

ج. لدي عادة تناول الطعام عندما أشعر بالملل، لكن في بعض الأحيان، يمكنني استخدام أنشطة أخرى بهدف عدم التفكير بالطعام.

د. لدي عادة كبيرة لتناول الطعام عندما أشعر بالملل. لا شيء يساعدني على كسر هذه العادة.

# 5

ا. أنا عادة ما أكون جائعا جسديا عندما آكل شيئا.

ب.أحيانًا، أتناول شيئًا ما بطريقة اندفاعية رغم عدم شعوري بالجوع حقا.

ج. لدي عادة تناول الطعام، والتي قد لا أستمتع بها حقا، لإرضاء شعور الجوع النفسي حتى لو لم أكن جائع جسديّا.

د. على الرغم من أنني لست جائعًا جسديًا ، ينتابني شعور بالجوع الذي لا يمكن تلبيته إلاّ بالأكل (سندويش). أحيانا ألجأ الى تقّيؤ الأكل حتى لا أكسب وزن.

# 6

ا. لا أشعر بأي شعور بالذنب أو الكراهية تجاه ذاتي بعد أن أفرط في تناول الطعام.

ب. بعد أن أفرط في تناول الطعام، أشعر أحيانًا بالذنب أو الكراهية تجاه ذاتي.

ج. أشكو من الشعور بالذنب أو الكراهية تجاه ذاتي تقريبا كل مرّة بعد أن أفرط في تناول الطعام.

# 7

ا. لا أفقد السيطرة الكاملة على طعامي عند اتباع نظام غذائي حتى بعد الإفراط في تناول الطعام لفترات معينة.

ب. في بعض الأحيان عندما أتناول "طعام ممنوع" ضمن نظام الغذاء، أشعر أنني أفسدته، الأمر الذي يؤدي الى تناول المزيد من الطعام.

ج. في كثير من الأحيان، أقول لنفسي " بما أني أفسدت نظامي الغذائي، فلأفسده حتى النهاية" عندما يحدث ذلك، آكل أكثر.

د. لدي عادة اعتياد اتباع نظام غذائي صارم لنفسي، لكني أخالف النظام الغذائي من خلال الأكل بشراهة. يبدو أن حياتي إما "وليمة" أو "مجاعة".

# 8

ا. نادراً ما آكل الكثير من الطعام الذي يشعرني بأني محشوّ بشكل غير مريح.

ب. عادة، وأنا أكل كمية كبيرة من الطعام مرة واحدة في الشهر، الأمر الذي يؤدي في نهاية المطاف الى شعوري بالحشو المفرط.

ج. لدي فترات منتظمة خلال الشهر عندما أتناول كميات كبيرة من الطعام، سواء في وقت الطعام أو في الوجبات الخفيفة.

د. أنا أكل الكثير من الطعام لدرجة أنني أشعر بعدم الارتياح بعد تناول الطعام و في بعض الأحيان بالغثيان قليلا.

# 9

ا. مستواي من السعرات الحرارية لا يرتفع كثيرا أو ينخفض كثيرا بشكل منتظم.

ب. في بعض الأحيان بعد أن أفرط في تناول الطعام، أحاول تقليل كمية السعرات الحرارية إلى ما يقارب "الصفر". لتعويض السعرات الحرارية الزائدة التي أكلتها.

ج. لدي عادة الإفراط بتناول الطعام خلال الليل. ليست الفكرة أنني سأكون جائعا في الصباح ولكن هذا هو روتيني.

د. في سنوات الرشد ، كانت لديّ فترات أسبوعية حيث كنت أحرم نفسي عمليّا من الأكل، يتبعها فترات من الأكل المفرط. يبدو أنني أعيش حياة إما "وليمة أو مجاعة."

#10

ا. أنا عادة أكون قادر على التوقف عن الأكل عندما أريد.

ب. في كل مرة أشعر برغبة في تناول الطعام، لا أستطيع السيطرة على هذه الرغبة.

ج. في كثير من الأحيان، أشعر برغبة قوية في تناول الطعام ،لا أستطيع السيطرة على هذه الرغبة، ولكن في أوقات أخرى أستطيع التحكم بدوافعي الغذائية.

د. أشعر أنني عاجز عن السيطرة على دوافعي الغذائية. لدي خوف من عدم القدرة على التوقف عن الأكل من تلقاء ذاتي.

# 11

ا. ليس لدي أي مشكلة في التوقف عن تناول الطعام عندما أشعر بالشبع.

ب. أنا عادة يمكن أن أتوقف عن الأكل عندما أشعر بالشبع ولكن أحيانا أفرط في تناول الطعام الأمر الذي يؤدي للشعور بالحشو الغير المريح .

ج. لدي مشكلة في التوقف عن الأكل عندما أبدأ وعادة ما أشعر بالحشو الغير المريح بعد أن آكل وجبة.

د. لأنه لدي مشكلة عدم القدرة على التوقف عن الأكل عندما أريد، أنا في بعض الأحيان أحثّ نفسي على التقيؤ لتخفيف شعوري بالحشو.

# 12

ا. يبدو أنني آكل عندما أكون مع الآخرين (العائلة والتجمعات الاجتماعية) بنفس الطريقة عندما أكون لوحدي.

ب. في بعض الأحيان، عندما أكون مع أشخاص آخرين، لا آكل بقدر ما أريد لأنه لديّ الوعي الذاتي حول طعامي.

ج. في الكثير من الأحيان، لا آكل سوى كمية صغيرة من الطعام عند وجود الآخرين، لأنني محرج للغاية بشأن أكلي.

د. أشعر بالخجل من الإفراط في تناول الطعام لدرجة أنني أختار الأوقات للإفراط في الأكل عندما أعرف إنه لن يراني أحد.

# 13

ا. أنا أتناول ثلاث وجبات في اليوم مع وجبة خفيفة (Snack ) أحيانا .

ب. أتناول 3 وجبات في اليوم، لكنني عادة أتناول وجبة خفيفة (Snack ) بين الوجبات.

ج. عندما أتناول وجبات خفيفة بشكل كبير، ألجأ الى تخطي الوجبات العادية.

د. هناك فترات عديدة تتّسم بعدم توقفي عن الأكل .

# 14

ا. لا أفكر كثيراً في محاولة السيطرة على الحوافز الغير المرغوبة في تناول الطعام.

ب. أحيانا، أشعر أن أفكاري مشغولة مسبقاً بمحاولة السيطرة على دوافعي الغذائية.

ج. أشعر أني أقضي الكثير من الوقت في التفكير في الكمية التي أكلتها أو التي أحاول عدم تناولها بعد الآن .

د. يبدو لي أن معظم أوقاتي مشغولة مسبقاً بالأفكار عن الأكل أو عدم الأكل. أشعر وكأنني أجاهد لعدم تناول الطعام.

#15

ا. أنا لا أفكر في الطعام كثيرا.

ب. لدي رغبة قوية في الحصول على الطعام ولكنها تدوم لفترات قصيرة فقط.

ج. لديّ أيام لا أستطيع فيها التفكير في أي شيء آخر سوى الطعام.

د. يبدو أن معظم أيام حياتي مشغولة بأفكار حول الطعام. أشعر أنني أعيش للأكل.

# 16

ا. أعلم عادةً ما إذا كنت جائعًا جسديًا أم لا. أتناول الكمية اللازمة من الطعام لإرضاء ذاتي .

ب. أشعر أحيانًا بعدم اليقين بشأن معرفة ما إذا جائعا جسديًا أم لا. في هذه الأوقات، من الصعب معرفة كمية الطعام التي يجب أن أتناولها لإرضائي.

ج. على الرغم من أنني قد أعرف عدد السعرات الحرارية التي يجب أن أتناولها، ليس لدي أي فكرة حول كمية الطعام الطبيعية التي أحتاجها.

**Appendix 2.**

**The Arabic version of the Hamilton Anxiety Rating Scale (HAM-A)**

| فيما يلي قائمة من العبارات التي تصف شعور معين لدى الناس. قيّم معدل االمريض من خلال إيجاد الجواب الذي يقدم أفضل وصف للحالة التي لديه. قم باختيار واحد من الأجوبة الخمسة لكل من الأسئلة الأربعة عشر. | | | | | |
| --- | --- | --- | --- | --- | --- |
|  | لا يوجد | معتدل | متوسط | شديد | شديد جدا |
| 1. مزاج قلق: هموم، توقّع الأسوأ، شعور سابق للخوف، تهيّج مفرط |  |  |  |  |  |
| 1. التوتر : مشاعر التوتر، تعب، إجفال ، البكاء بسهولة، رجفة ، مشاعر التملّل وعدم القدرة على الاسترخاء |  |  |  |  |  |
| 1. المخاوف: من الظلام، من الغرباء، من الوحدة، من الحيوانات، من إزدحام السير، من الحشود |  |  |  |  |  |
| 1. الأرق :صعوبة الخلود الى النوم، نوم متقطع، فترة نوم غير مرض والشعور بالتعب عند الإستيقاظ، والأحلام والكوابيس الرعب الليلي |  |  |  |  |  |
| 1. الحالة الذهنية: صعوبة في التركيز وضعف الذاكرة |  |  |  |  |  |
| 1. مزاج مكتئب : فقدان الاهتمام، وعدم المتعة في ممارسة الهوايات، والاكتئاب، والاستيقاظ المبكر، و تقلّبات المزاج خلال النهار |  |  |  |  |  |
| 1. حالة جسدية (عضلية): الآلام والأوجاع، والوخز، وصلابة، إنكماش سريع في العضلات، صوت متقلب، زيادة حجم العضلات. |  |  |  |  |  |
| 1. حالة جسدية (حسيّة): طنين، وعدم وضوح الرؤية، هبّات ساخنة وباردة، ومشاعر بالضعف،إحساس بالوخز |  |  |  |  |  |
| 1. عوارض القلب والأوعية الدموية: عدم انتظام في دقات القلب، والخفقان، وألم في الصدر، والخفقان في الأوعية القلبية، شعور بالإغماء، نبض متقطّع وناقص. |  |  |  |  |  |
| 1. عوارض الجهاز التنفسي: ضغط أو انقباض في الصدر شعور بالاختناق ، تنهد، وضيق التنفس. |  |  |  |  |  |
| 1. عوارض الجهاز الهضمي: صعوبة في البلع، وجع وآلام في البطن، وإحساس بالحرق، امتلاء البطن، والغثيان، والتقيؤ، قرقرة الأمعاء ر، رخاوة في الأمعاء (عدم السيطرة على التغوط)، وفقدان الوزن، والإمساك. |  |  |  |  |  |
| 1. عوارض في جهاز التناسلي :كثرة التبويل ، إلحاح التبول، وانقطاع الطمث، وغزارة الطمث، فتور جنسي ، قذف مبكر ، وفقدان الرغبة الجنسية والعجز الجنسي |  |  |  |  |  |
| 1. أعراض الجهاز العصبي الذاتي واللإرادي :جفاف الفم واحمرار الوجه، شحوب الوجه ، الميل للتعرّق ، دوار، صداع ناتج عن الضغط ، وزيادة الشعر. |  |  |  |  |  |
| 1. السلوك خلال المقابلة: تململ بحركات عصبية عدم البقاء بمكانه، و، وإرتعاش الأيدي، تقطب الجبين ، وجه متوتر أو منقبض، تنهد أو تنفس سريع، وشحوب الوجه، بلع متواصل ، الخ |  |  |  |  |  |

**Appendix 3.**

**The Arabic version of the Hamilton Scale for Depression (HAM-D)**

يرجى إختيار الجواب الأقرب الذي يدل على شدة الأعراض خلال الأسبوع الماضي

1**- مزاج إكتئابي (حزن، يأس، عجز وانعدام القيمة)**

0 = لا يوجد 1 = موقف كئيب، تشاؤم، يأس 2 = بكاء متقطع

3 = بكاء متكرر 4 = يصرّح المريض فقط بتلك المشاعر من خلال تعابيره اللفظية والغير لفظية

2**- الشعور بالذنب**

0= لا يوجد 1= لوم النفس، يشعر إنه يخذل الأشخاص

2= أفكار بالذنب أو /تفكير متكرّر بأخطاء سابقة من الماضي أو أفعال خاطئة

3 = المرض الحالي هو نوع من العقاب. أوهام بالشعور بالذنب

4 = يسمع أصوات معادية أو إتهامية و/ أو إجتاز فترات من الهلوسة البصريّة المبنيّة على التهديد . أوهام بالشعور بالذنب

**3- الانتحار**

0= لا يوجد 1= الشعور بأن الحياة لا تستحق أن تعاش 2= يتمنى لو انه قد مات أو أي أفكار من احتمال وفاته

3= أفكار انتحارية 4= محاولات إنتحارية (أي محاولة جديّة للانتحار تقاس بدرجة 4 )

**4- الأرق المبكر**

0= لا يوجد صعوبة في النوم 1= يشكو من صعوبة في بعض الأحيان في النوم، أي بمعنى، أكثر من 1/2 ساعة

2= يشكو من صعوبة في النوم ليلا

**5- أرق في منتصف النوم**

0= لا يوجد أي صعوبة 1= المريض يشكو من كونه قلق ومنزعج أثناء الليل

2= الاستيقاظ خلال الليل: عندما يترك الفرد السرير ضع علامة 2 (باستثناء الدخول للمرحاض)

**6- الأرق المتأخر**

0= لا يوجد صعوبة 1= الاستيقاظ في الساعات الأولى من الصباح، ثمّ العودة الى النوم

2 = عدم القدرة على النوم مرة أخرى إذا ترك الفرد سريره

**7- العمل والأنشطة**

0= لا يوجد صعوبة 1= أفكار ومشاعر مرتطبة بالعجز، بالتعب أو ضعف متعلق بالنشاطات : العمل أو الهوايات

2 = فقدان الاهتمام بالنشاطات ؛ بالهوايات أو بالعمل. وقد تعبّر هذه الصعوبات بطريقة مباشرة أو غير مباشرة من خلال فتور الهمة، والحيرة والتردد (يشعر أنه يجب دفعه للقيام بالعمل أو الأنشطة).

3 = نقص في الوقت الفعلي الذي يقضيه في الأنشطة أو في النشاطات الإنتاجية

4 = توقف عن العمل بسبب الحالة المرضيّة

**8- التأخّر : الحركي (بطء التفكير والكلام، ضعف القدرة على التركيز، وانخفاض النشاط الحركي)**

0 = التكلّم بطريقة عادية ووجود أفكار عادية 1 =تأخّر طفيف خلال المقابلة

2 = تأخّر واضح خلال المقابلة العياديّة 3 = صعوبة في مباشرة المقابلة العياديّة 4 = ذهول كامل

**9- الحركة الدائمة (إهتياج)**

0 = لا يوجد 1 = الشعور بالإثارة يعبّر عنها بحركة مستمرّة 2 = اللعب في اليدين والشعر، الخ

3 = حركة مفرطة، لا يمكنه أن يبقى جالسا 4 = فرك اليدين، قضم الأظافر، شد الشعر، عض الشفاه

**10- القلق (النفسي)**

0= لا صعوبة 1 = توتر ذاتي وتهيج 2 = القلق بشأن أمور صغيرة

3 = موقف قلقي واضح من خلال تعابير الوجه أوالحوار 4 = مخاوف معبّر عنها دون أي استجواب

**11- القلق الجسدي: القلق الجسدي المتتالي، (نشاط اللاإرادي مفرط، عسر الهضم ومغص في البطن والتجشؤ والاسهال، والخفقان، وفرط التنفس، تنمل، والتعرق واحمرار الوجه، الارتعاش، الصداع، وتكرار البول). تجنب السؤال عن الآثار الجانبية المحتملة للدواء (مثلاً جفاف الفم، والإمساك)**

0= لا يوجد 1= معتدل 2= متوسط 3= شديد 4= شديد جدا

**12- الأعراض الجسدية – (مشاكل في الجهاز الهضمي)**

0= لا يوجد

1= فقدان الشهية ولكن تناول الطعام دون التشجيع من الآخرين. تناول الطعام بشكل طبيعي

2 = صعوبة في تناول الطعام دون حث من الآخرين. انخفاض ملحوظ في الشهية وتناول الطعام

**13- أعراض جسدية بشكل عام**

0= لا يوجد

1= ثقل في الأطراف والظهر أو الرأس. آلام الظهر، والصداع، وآلام في العضلات. فقدان الطاقة وتعب

2 = أي أعراض واضحة المعالم تقاس بمعدّل 2

**14- الأعراض التناسلية ( مثل: فقدان الرغبة الجنسية، ضعف الأداء الجنسي، اضطرابات الدورة الشهرية)**

0= لا يوجد 1= معتدل 2= شديد

**15- المرض الوهمي**

0 = غير موجود 1 = إمتصاص ذاتي (جسدي) 2 = الانشغال بالصحة

3 = الشكاوى المتكررة، وطلب متكرّر للحصول على المساعدة، الخ 4 = أفكار هذيانية

**16-فقدان الوزن**

0 = لا خسارة في الوزن 1 = فقدان في الوزن ربما مرتبط بالمرض الحالي

2 = خسارة مؤكدة للوزن (حسب المريض) 3 = لم يتمّ التقييم

**17- المعرفة (البصيرة)**

0 = الإعتراف بأنه كئيب وعنده إضطراب

1 = يقرّ بالمرض ولكنه ينسب الأسباب لرداءة الطعام والمناخ والإرهاق، والفيروسات، والحاجة للراحة، الخ

2 = ينفي كلّياً كونه مريض

**Appendix 4.**

**The Arabic version of the Dutch Restrained Eating Scale**

| **Dutch Restrained Eating Scale** | | | | | |
| --- | --- | --- | --- | --- | --- |
|  | **أبدا** | **نادرا** | **أحيانا** | **في كثير من الأحيان** | **كثيرا جدا** |
| 1. عندما يزيد وزنك، هل تأكل أقل مما تفعله عادة؟ |  |  |  |  |  |
| 1. في أوقات الوجبات هل تحاول أن تأكل أقل مما ترغب ؟ |  |  |  |  |  |
| 1. كم مرة ترفض الطعام أو الشراب المقدم لأنك تشعر بالقلق تجاه وزنك؟ |  |  |  |  |  |
| 1. هل تتابع بدقة ما تأكله؟ |  |  |  |  |  |
| 1. هل تتعمّد تناول الأطعمة التي تنحّف؟ |  |  |  |  |  |
| 1. إذا كنت قد أكلت كثيراً في وجبة معينة ، هل تأكل أقل من المعتاد في اليوم التالي؟ |  |  |  |  |  |
| 1. هل تتعمّد أن تأكل أقل لكي لا تصبح أثقل؟ |  |  |  |  |  |
| 1. كم مرة تحاول ٲن لا تٲكل بين وجبات الطعام لأنك تتابع وزنك؟ |  |  |  |  |  |
| 1. كم مرة في المساء تحاول عدم الٲكل لأنك تتابع وزنك؟ |  |  |  |  |  |
| 1. هل تأخذ بعين الاعتبار وزنك مع ما تأكله؟ |  |  |  |  |  |
